# Chemical Constituents of the Methanolic Extract of Leaves of *Leiothrix spiralis* Ruhland and Their Antimicrobial Activity

**DOI:** 10.3390/molecules161210479

**Published:** 2011-12-16

**Authors:** Marcelo Gonzaga de Freitas Araújo, Felipe Hilário, Leonardo Gorla Nogueira, Wagner Vilegas, Lourdes Campaner dos Santos, Taís Maria Bauab

**Affiliations:** 1 Department of Biological Sciences, Faculty of Pharmaceutical Sciences, São Paulo State University-UNESP, 14801-902, Araraquara, SP, Brazil; 2 Department of Organic Chemistry, Chemistry Institute, São Paulo State University-UNESP, 14800-900, Araraquara, SP, Brazil

**Keywords:** Eriocaulaceae, *Leiothrix spiralis*, phenolic compounds, antimicrobial activity

## Abstract

Chemical fractionation of the methanolic extract of leaves of *Leiothrix spiralis* Ruhland afforded the flavonoids luteolin-6-*C*-β-D-glucopyranoside (**1**), 7-methoxyluteolin-6-*C*-β-D-glucopyranoside (**2**), 7-methoxyluteolin-8-*C*-β-D-glucopyranoside (**3**), 4′-methoxyluteolin-6-*C*-β-D-glucopyranoside (**4**), and 6-hydroxy-7-methoxyluteolin (**5**), and the xanthones 8-carboxymethyl-1,5,6-trihydroxy-3-methoxyxanthone (**6**), 8-carboxy-methyl-1,3,5,6-tetrahydroxyxanthone (**7**). Methanolic extract, fractions, and isolated compounds of the leaves of *L. spiralis* were assayed against Gram-positive (*Staphylococcus aureus*, *Bacillus subtilis *and *Enterococcus faecalis*) and Gram-negative bacteria (*Escherichia coli*, *Pseudomonas aeruginosa*, *Salmonella setubal *and *Helicobacter pylori*) and fungi (the yeasts *Candida albicans*, *C. tropicalis*, *C. krusei* and *C. parapsilosis*). We observed the best minimum inhibitory concentration values for the methanolic extract against *Candida parapsilosis*, for the fraction **5** + **6** against Gram-negative bacteria *E. coli* and *P. aeruginosa*, and compound **7** against all tested *Candida* strains. The methanolic extract contents suggest that this species may be a promising source of compounds to produce natural phytomedicines.

## 1. Introduction

The Eriocaulaceae is a pantropical family which consists of 11 genera and about 1,200 species. The genus *Leiothrix* belongs to the Eriocaulaceae family and is exclusively South American, including about 37 species mainly restricted to Brazil [1]. All species of *Leiothrix *subg. *Stephanophyllum* are pseudoviviparous, and some of them (*L. spiralis* and *L. vivipara*) are endemic to the state of Minas Gerais, Brazil. This specie is a frequent component of the vegetation in pools of hilly areas or swamps, especially in sandy ground areas, with low water retention [2]. Little is known about the ethnopharmacological property of Eriocaulaceae. Literature reports the traditional use of Eriocaulaceae species to treat skin ulcers and bacterial infections [3], and some studies have described the antioxidant [4], cytotoxic, mutagenic [5,6], and antiulcerogenic activities [7]. Meanwhile, some species of this family are of great economic importance since they are exported as ornamental plants to various countries, mainly Germany and Japan [8].

Chemical studies on *Leiothrix* species are also scarce. The presence of soluble phenolic compounds with antimicrobial activity has been investigated in ramets of 21 *Leiothrix *species, with identification of nepetin-7-*O*-β-D-glucopyranoside, nepetin-7-*O*-β-D-arabinopyranoside, and luteolin *O-* and *C-*glucopyranoside [9,10]. Another study has determined the presence of xanthones and flavone with antioxidant activity in ramets/flower heads of *L. flavescens *and *L. curvifolia *[11]. Therefore, we performed a phytochemical study of methanolic extract of the *L. spiralis* leaves and investigated the antimicrobial activity of the extract, fractions and isolated compounds on different strains of bacteria and yeasts*.*

## 2. Results and Discussion

**Figure 1:**
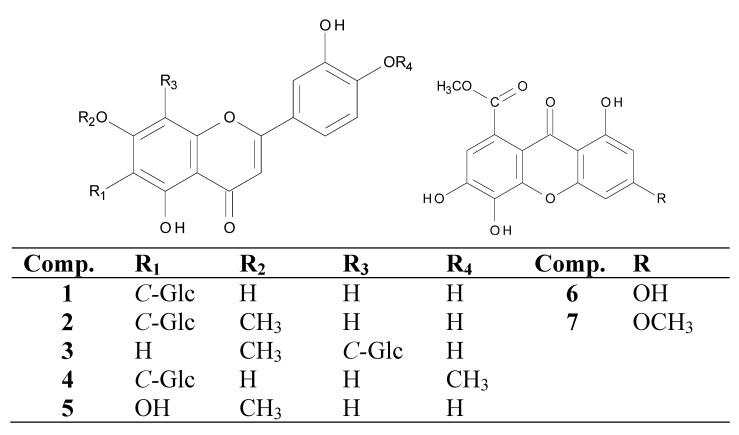
Compounds isolated from the MeOH extract of leaves of *L. spiralis*.

The structures of isolated compounds **1–7** were determined by spectroscopic analysis (1D-, 2D-NMR, UV, ES-MS) and by comparison of spectral data to related metabolites (Figure 1). The chemical constituents of Eriocaulaceae are of taxonomic importance. Naphthopyranones and flavonols are the major compounds in many *Paepalanthus *species [9,12], but are absent in species belonging to both the *Syngonanthus* and the *Leiothrix* genus [2,9,13,14].

In this study, five flavone and two xanthones were detected in a methanolic extract of *L. spiralis*. The presence of flavones in *L. spiralis* corroborates the previously reported data [9]. Xanthones **6** and **7** have been isolated from *L. curvifolia* and *L. flavescens* [7,13] and present antioxidant activity [11]. The presence of a COOCH_3_ group at C-8 of the xanthone ring is unusual, since most xanthones isolated from higher plants presents a methyl group at C-3, while the xanthones isolated from lichens have a methyl group at C-8 position [15]. Previous investigators have proposed that lichen xanthones are biosynthesized by cyclization of a single, linear polyketide chain [16]. It has been proposed that *Leiothrix* xanthones are biosynthetically related to naphthopyranones isolated from species of the *Paepalanthus* genus (Eriocaulaceae) [12].

Minimum inhibitory concentration (MIC) analysis revealed antibacterial and antifungal activity of the almost all tested sample (Table 1). Holetz *et al*. [17] considered that if the extracts displayed an MIC less than 100 µg/mL, the antimicrobial activity was good; from 100 to 500 µg/mL the antimicrobial activity was moderate; from 500 to 1,000 µg/mL the antimicrobial activity was weak; over 1,000 µg/mL the extract was considered inactive. Based on these criteria, the extract presented described that MIC concentrations higher than 100 µg/mL of phytochemical weak activity against Gram-positive bacteria, and moderate activity against *C. parapsilosis*. Gibbons [18] compounds would not be expected to have clinical signiﬁcance. MIC results show that Fraction **5** + **6** and compound **7** were able to prevent the growth of some tested bacterial species. The fraction **5** + **6** showed the lowest MIC value against *E. coli* (62.5 µg/mL) and *P. aeruginosa *(31.25 µg/mL)*.* This activity could be considered as promising, considering the fact that the tested bacteria are resistant to first line antibiotics, and also that *P. aeruginosa *is a leading cause of nosocomial infections [19,20].

Determination of minimum bacterial concentration (MBC) and minimum fungicidal concentration (MFC) (Table 1) confirmed that a cidal effect of most of the tested samples could be expected on the studied microorganisms. As can be observed, most of the significant MBC and MFC values are similar to the corresponding MIC values, mainly in yeasts.

All tested samples were found to be active against at least one of *Candida *species. Compound **7** had the best result against all tested yeast species. Previous studies have reported some xanthone derivatives as remarkable antifungal agents. The antifungal profile of the described xanthones suggests that, in the majority of cases, hydroxyl groups are important for activity [21,22]. It was reported as likely that antifungal xanthones from plants required three or four hydroxyl groups, in which one or two of them must be at C-5 and/or C-6, and a hydrophobic group must be on one of the aromatic rings, and compound **7** meets these requirements [23]. We demonstrated that compound **7** exhibits fungicidal activity against *C. krusei* and *C. parapsilosis*. According to the literature, in general, xanthones have fungicidal rather than fungistatic activity. The putative mechanism pointed to the cell membrane as a possible target, by hypothesizing that xanthones act by the inhibition of ergosterol biosynthesis [24]. The antifungal activity of the phenolic compounds has been attributed to their lipophilic properties, which determine their ability to penetrate into the plasma membrane and induce changes in the physico-chemical properties of the cell wall, cell membrane, and cellular organelles. Another hypothesis include inhibition of enzimes produced by fungi and authors have linked the antioxidant activity of phenolic compounds to their activity on the biosynthesis inhibition of these metabolites [25]. The strong *in-vitro* antifungal activity of xanthone suggests that this compound might have wide pharmaceutical use.

**Table 1:** Antibacterial and antifungal activity of methanolic extract, fractions, and compounds isolated from *L. spiralis* leaves.

Tested samples	MIC (MBC)^a^						MIC (MFC) ^a^				
	Gram-positive bacteria			Gram-negative bacteria				Fungi			
	*S. aureus*	*B. subtilis*	*E. faecalis*	*E. coli*	*P. aeruginosa*	*S. setubal*		*C. albicans*	*C. krusei*	*C. parapsilosis*	*C. tropicalis*
Extract	1000 (1000)	1000 (1000)	500 (-)	-	-	-		1000 (1000)	500 (1000)	250 (250)	1000 (1000)
Fraction (**5** + **6**)	125 (250)	250 (250)	-*	62.5 (250)	31.25 (250)	-*		-*	-*	125 (125)	-*
Fraction (**2** + **3**)	-*	-*	-*	-*	-*	-*		-*	250 (250)	125 (125)	250 (250)
(**1**)	-*	-*	-*	-*	-*	-*		-*	-*	125 (125)	-*
(**4**)	-*	-*	-*	-*	-*	-*		125 (250)	-*	-*	-*
(7)	125 (-*)	125 (125)	-*	-*	125 (250)	-*		62.5 (250)	15.7(15.7)	15.7 (15.7)	31.25 (62.5)
Positive controls ^b^	2.1 × 10^−2^	2.1 × 10^−2^	2.1 × 10^−2^	3.3 × 10^−5^	3.3 × 10^−5^	3.3 × 10^−5^		32 (>64)	8 (>64)	8 (>64)	32 (>64)
	(2.1 × 10^−2^)	(2.1 × 10^−2^)	(2.1 × 10^−2^)	(3.3 × 10^−5^)	(3.3 × 10^−5^)	(3.3 × 10^−5^)					

^a^ Values given as µg/mL. ^b^ ciprofloxacin for bacteria and fluconazole for fungi (-) MIC/MBC/MFC > 1000 µg/mL; (-*) MIC/MBC/MFC > 500 µg/mL. MIC: minimum inhibitory concentration; MBC/MFC: minimum bactericidal/fungicial concentration.

All other samples had no significant activity. Despite of the phenolic content, the extract showed weak activity against Gram-positive bacteria. However, these findings suggest that phenols, mainly xanthones, are among the chemical classes responsible for the antibacterial activity of *L. spiralis*. Fang *et al*. [26] found that five phenols, among then xanthones and flavones, isolated from *Eriocaulon buergerianum*, a Eriocaulaceae specie, exhibited potential antibacterial activity against *S. aureus*. The flavones of Fraction **2** + **3** showed moderate activity against *Candida* species. Previous investigators have also documented the antibacterial and antifungal properties of flavonoids [26,27,28,29]. This activity might be explained by their ability to complex with cell wall and induce the formation of pseudomulticellular aggregates, inhibiting microbial growth [30,31]. Methanolic extract of leaves of *L. spiralis* inhibited weakly the growth of all yeasts tested. Silva *et al*. [32] have reported the anti-*C. albicans *activity of this extract. The MIC values observed by us are similar to those reported by Silva *et al*. [32]; however, the activity was observed in the present study against other *Candida *species.

Activity against *H. pylori* of the extract and compounds tested was not evident under the present experimental conditions, and zones of inhibition were not produced. In this study, the bacteria was susceptible to clarithromycin (positive control), whose diameter of inhibition zone was 21.8 ± 1 mm. A previous study has shown anti-*H. pylori* activity of the methanolic extract of *L. flavescens*. Such activity can be correlated with the presence of flavones and xanthones with a catechol nucleus in their structures [33]. Despite the fact that most of the compounds detected in methanolic extract of leaves of *L. spiralis* were flavones and xanthones, we used a different methodology and did not observe activity against *H. pylori.*

## 3. Experimental

### 3.1. General Procedures

Melting point was measured on a MQ APF-301 (Microquímica^®^, Brazil) digital apparatus. UV spectrum was recorded on a HACH UV-Vis DR/4000 spectrophotometer in MeOH [34]. IR spectrum was obtained using Shimadzu FT–IR 8300 spectrophotometer in KBr disk. NMR analyses and 2D experiments were run on Varian^®^ INOVA 500 operating at 500 MHz for ^1^H and 125 MHz for ^13^C (11.7 T), using TMS as internal standard. ESIMS in negative and positive ion mode and mass spectra were acquired and processed using the software (version 1.3) provided by the manufacturer. The capillary voltage was set at −32 V, the spray voltage at 5 kV and the tube lens offset at 30 V. Capillary temperature was 280 °C. Each compound was dissolved in CH_3_OH and infused in the ESI source by using a syringe pump (flow rate 5 µL/min). TLC analyses were performed on silica gel 200 μm (Sorbent Technologies^®^) and visualized using UV light (254 and 365 nm).

### 3.2. Plant Material

Leaves of *L. spiralis* were collected in May 2006 in Diamantina, state of Minas Gerais, Brazil and authenticated by Dr. Paulo Takeo Sano from the Institute of Biosciences of the University of São Paulo (IB-USP), São Paulo. A voucher specimen (SANO No. 4798) was deposited at the IB-USP Herbarium.

### 3.3. Extraction and Isolation

Leaves of *L. spiralis *were dried in an oven at 45 °C for one week. The dried leaves of *L. spiralis* (2.00 g) were powdered and extracted successively with hexane, methylene chloride (500 mL each solvent) by maceration at room temperature (one week each solvent) and the methanol (500 mL) were extracted by percolation (one week). The solvents were removed under vacuum to yield the crude extracts. The methanol extract (1.2 g) was chromatographed on a Sephadex LH-20 column (80 × 2 cm), with MeOH as eluent.

Fractions (8 mL) were collected and checked by TLC [Si gel plates, CHCl_3_/MeOH/*n*-PrOH/H_2_O, (5:6:1:4, v/v/v/v)]. Fractions **79**–**82** (70 mg) were further purified by medium pressure liquid chromatography (MPLC) using a column of RP-18 silica gel (9.0 cm × 1.5 mm i.d.) eluted with MeOH H_2_O (5:95 v/v, initial, after gradient until methanol), affording 4'-methoxyluteolin-6-*C*-α-L-glucopyranoside (**4**, 8.0 mg) [35], 8-carboxymethyl-1,3,5,6-tetrahydroxyxanthone (**7**, 7.0 mg), 8-carboxymethyl-1,5,6-trihydroxy-3-methoxyxanthone (**6**, 9.0 mg) [13], and 6-hydroxy-7-methoxy-luteolin (**5**, 10.0 mg) [36]. Fractions **61**–**68** (295 mg) were further purified by MPLC using the same conditions described for fractions **79**–**82**, yielding 7-methoxyluteolin-6-*C*-β-D-glucopyranoside (**2**, 12.0 mg) [37] and 7-methoxyluteolin-8-*C*-β-D-glucopyranoside (**3**, 15.0 mg) [38]. Fractions **69**–**74** (30 mg) were further purified by MPLC using the same conditions indicated for fractions **79**–**82**, yielding luteolin-6-*C*-β-D-glucopyranoside (**1**, 10.0 mg) [39]. Although all the compounds have been identified separately, compounds **2** and **3** were isolated in a single fraction, as well as the compounds **5** and **6**, thus named Fraction (**2** + **3**) and Fraction (**5** + **6**), respectively.

*Luteolin-6-C-β-D-glucopyranoside *(**1**): UV λ_max_ nm: (MeOH): 260, 288 e 360. ES-MS (positive mode) *m/z *449 [M+H]^+^. ^1^H-NMR (DMSO-*d6*): *δ* 7.41 (1H, dd, *J* = 8.0, 1.5 Hz, H-6′), 7.40 (1H, dd, *J* = 1.5 Hz, H-2′), 6.88(1H, d, *J* = 8.0 Hz, H-5′), 6.68 (1H, s, H-8), 6.46 (1H, s, H-3), 4.61 (1H, d, *J* = 10.0 Hz, H-1′′-Glc), 4.00 (2H, dd, 9.0, 9.0, H-2′′-Glc), 3.70 (1H, dd, 5.5, 6.0, H-6b′′-Glc), 3.37 (1H, dd, 5.5, 6.0, H-6a′′-Glc), 3.21 (2H, d, 9.0, H-3′′-Glc), 3.16 (1H, m, H-5′′-Glc), 3.12 (1H, m, H-4′′-Glc). ^13^C-NMR (DMSO-*d6*):181.0 (C, C-4), 163.7 (C, C-2-7), 160.0 (C, C-5), 156.0 (C, C-9), 154.6 (C, C-4′), 146.0 (C, C-3′), 121.6 (C, C-1′), 119.0 (CH, C-6′), 115.0 (CH, C-5′), 113.8 (CH, C-2′), 109.0 (C, C-6), 104.0 (C, C-10), 102.9 (CH, C-3), 93.5 (CH, C-8), 81.2 (CH, C-5′′-Glc), 78.2 (CH, C-3′′-Glc), 73.0 (CH, C-1′′-Glc), 70.4 (CH, C-2′′-Glc), 70.8 (CH, C-4′′-Glc), 61.8 (CH_2_, C-6′′-Glc).

*7-Methoxyluteolin-6-C-β-D-glucopyranoside *(**2**): UV λ_max_ nm: (MeOH): 254, 270 e 349 nm. ES-MS (negative mode): *m/*z 461 [M−H]^−^, 371 [M−90−H]^−^, 341 [M−120−H]^−^. ^1^H-NMR (DMSO-*d6*): *δ* 7.46 (1H, d, *J* = 1.5 Hz, H-2`), 6.88 (1H, d, *J* = 8.0 Hz, H-5′), 6.79 (1H, s, H-8), 6.75 (1H, s, H-3), 6.48 (1H, dd, *J* = 8.0, 1.5 Hz, H-6′), 3.89 (3H, s, 7-OCH_3_), 4.57 (1H, d, *J* = 10.0 Hz, H-1′′ Glc), 3.48 dd (1H, dd, 9.0, 9.0, H-4′′-Glc), 3.70 (1H, dd, 3.5, 12.0, H-6a′′-Glc), 3.38 (1H, dd, 4.5, 12.0, H-6b′′-Glc), 3.16 (1H, dd, 10.5, 9.5, H-3′′-Glc), 3.18 (1H, m, H-5′′-Glc), 3.12 (1H, 9.0, 7.5, H-2′′-Glc). ^13^C-NMR (DMSO-*d6*): 181.0 (C, C-4), 163.9 (C, C-2), 163.7 (C, C-7), 156.9 (C, C-9), 156.6 (C, C-5), 149.9 (C, C-4′), 145.9 (C, C-3′), 121.4 (C, C-1′), 118.3 (CH, C-6′), 115.6 (CH, C-5′), 112.8 (CH, C-2′), 109.6 (C, C-6), 104.3 (C, C-10), 103.0 (CH, C-3), 90.5 (CH, C-8), 81.9 (CH, C-5′′-Glc), 78.2 (CH, C-3′′-Glc), 72.8 (CH, C-1′′-Glc), 70.4 (CH, C-2′′-Glc), 71.4 (CH, C-4′′-Glc), 61.4 (CH_2_, C-6′′ Glc), 56.5 (C-7-OCH_3_). 

*7-Methoxyluteolin-8-C-β-D-glucopyranoside *(**3**): UV λ_max_ nm: (MeOH): 258, 288 e 356. ES-MS (negative mode) *m/z *461 [M−H]^−^, 371 [M−90−H]^−^, 341 [M−120−H]^−^. ^1^H-NMR (DMSO-*d6*): *δ* 7.54 (1H, dd, *J* = 8.0, 1.5 Hz, H-6′), 7.48 (1H, d, *J* = 1.5 Hz, H-2′), 6.86 (1H, d, *J* = 8.0 Hz, H-5′), 6.68 (1H, s, H-3), 6.50 (1H, s, H-6), 3.87 (3H, s, 7-OCH_3_), 4.70 (1H, d, *J* = 10.0 Hz, H-1′′-Glc), 3.82 (1H, dd, 9.0, 9.5, H-4′′-Glc), 3.68 (1H, dd, 3.5, 12.0, H-6a′′-Glc), 3.40 (1H, dd, 8.0, 7.5, H-3′′-Glc), 3.34 (1H, dd, 4.5, 12.0, H-6b′′-Glc), 3.24 (1H, dd, 8.5, 9.0, H-2′′-Glc), 3.14 (1H, m, H-5′′-Glc). ^13^C-NMR (DMSO-*d6*):181.0 (C, C-4), 163.8 (C, C-7), 164.4 (C, C-2), 161.1 (C, C-5), 155.1 (C, C-9), 149.7 (C, C-4′), 145.8 (C, C-3′), 121.6 (C, C-1′), 119.1 (CH, C-6′), 115.4 (CH, C-5′), 113.9 (CH, C-2′), 104.3 (C, C-8), 104.3 (C, C-10), 102.4 (CH, C-3), 94.9 (CH, C-6), 81.6 (CH, C-5′′-Glc), 78.2 (CH, C-3′′-Glc) 72.8 (CH, C-1′′-Glc), 70.2 (CH, C-4′′-Glc), 70.2 (CH, C-2′′-Glc), 61.4 (CH_2_, C-6′′- Glc), 56.5 (C-7-OCH_3_). 

*4′-Methoxyluteolin-6-C-β-D-glucopyranoside *(**4**): UV λ_max_ nm: (MeOH): 243, 271 e 342. ES-MS (positive mode) *m/z *463 [M+H]^+^. ^1^H-NMR (DMSO-*d6*): *δ* 7.40 (1H, d, *J* = 1.5 Hz, H-2′), 7.38 (1H, dd, *J* = 8.0, 1.5 Hz, H-6′), 6.88 (1H, d, *J* = 8.0 Hz, H-5′), 6.68 (1H, s, H-8), 6.46 (1H, s, H-3), 3.89 (3H, s, 4′-OCH_3_), 4.68 (1H, dd, *J* = 12.0, 2.5 Hz, H-6b′′-Glc), 4.61 (1H, d, *J* = 10.0 Hz, H-1′′-Glc), 4.36 (1H, dd, *J* = 12.0, 5.0 Hz, H-6a′′-Glc), 4.00 (1H, dd, *J* = 9.0, 9.0 Hz, H-2′′-Glc), 3.21 (1H, dd, *J* = 9.0, 9.0 Hz, H-3′′-Glc), 3.18 (1H, dd, *J* = 9.0, 9.0 Hz, H-4′′-Glc), 3.15 (1H, m, H-5′′-Glc), 3.23 (1H, dd, *J* = 9.0, 9.0 Hz, H-4 Ara), 3.21 (1H, dd, *J* = 9.0, 9.0 Hz, H-3 Ara). ^13^C-NMR (DMSO-*d6*): 181.0 (C, C-4), 163.4 (C, C-7), 160.0 (C, C-5), 154.6 (C, C-4′), 156.0 (C, C-9), 146.0 (C, C-3′), 121.6 (C, C-1′), 118.2 (CH, C-6′), 113.0 (CH, C-2′), 115.0 (CH, C-5′), 109.0 (C, C-6), 104.0 (C, C-10), 103.0 (CH, C-3), 93.6 (CH, C-8), 81.0 (CH, C-5′′-Glc), 78.6 (CH, C-3′′-Glc) 73.3 (CH, C-1′′-Glc), 70.8 (CH, C-4′′-Glc), 70.5 (CH, C-2′′-Glc), 61.0 (CH_2_, C-6′′-Glc), 56.0 (C7-OCH_3_). 

*6-Hydroxy-7-methoxyluteolin *(**5**): UV λ_max_ nm: (MeOH): 254, 273, 348, ES-MS (negative mode) *m/z *315 [M–H]^−^. ^1^H-NMR (DMSO-*d6*): *δ* 7.42 (1H, dd, *J* = 1.5 Hz, H-2′), 7.41 (1H, dd, *J* = 8.0, 1.5 Hz, H-6′), 6.89 (1H, d, *J* = 8.0 Hz, H-5′), 6.66 (1H, s, H-3), 6.84 (1H, s, H-8), 3.90 (3H, s, 4′-OCH_3_). ^13^C-NMR (DMSO-*d6*):182.1 (C, C-4), 163.8 (C, C-2), 155.6 (C, C-7), 152.0 (C, C-9), 151.0 (C, C-5), 149.7 (C, C-4′), 145.8 (C, C-3′), 131.4 (C, C-6), 121.6 (C, C-1′), 119.0 (CH, C-6′), 115.6 (CH, C-5′), 113.4 (CH, C-2′), 102.4 (CH, C-3), 104.1 (C, C-10), 91.0 (CH, C-8), 56.0 (C7-OCH_3_).

*8-Carboxymethyl-1,5,6-trihydroxy-3-methoxyxanthone *(**6**): UV λ_max_ nm: (MeOH): 204, 252, 292, 325. EI-MS (positive mode) *m*/*z* 332 [M]^+^, 333 [M+H]^+^ 301 [M−OCH_3_]^+^, 273 [M−COOCH_3_]. ^1^H-NMR (DMSO-*d6*): δ 6.15 (1H, d, *J* = 1.5 Hz, H-2), 6.38 (1H, d, *J* = 1.5 Hz, H-4), 6.79 (1H, s, H-7), 3.79 (3H, s, 3-OCH_3_), ^13^C-NMR (DMSO-*d6*): *δ* 164.8 (C-1), 98.1 (C-2), 168.2 (C-3), 93.8 (C-4), 157.4 (C-4a), 146.7 (C-4b), 134.8 (C-5), 151.0 (C-6), 111.8 (C-7), 124.9 (C-8), 111.2 (C-8a), 180.0 (C-9), 102.7 (C-9a),171.4 (C-10), 56.4 (C-3-OCH_3_).

*8-Carboxymethyl-1,3,5,6-tetrahydroxyxanthone *(**7**): UV λ_max_ nm: (MeOH): 205, 244, 288, 315, 362. EI-MS (positive mode) *m/z* 346 [M]^+^, 315 [M−OCH3]^+^, 285 [M−2OCH3+H]^+^. ^1^H-NMR (DMSO-*d6*): *δ* 6.34 (1H, d, *J* = 1.5 Hz, H-2), 6.57 (1H, d, *J* = 1.5 Hz, H-4), 6.78 (1H, s, H-7), 3.87 (3H, s, 3-OCH3), ^13^C-NMR (DMSO-*d6*): *δ* 163.8 (C-1), 97.6 (C-2), 166.4 (C-3), 93.4 (C-4), 157.8 (C-4a), 146.0 (C-4b), 135.1 (C-5), 150.0 (C-6), 112.4 (C-7), 124.5 (C-8), 110.9 (C-8a), 179.0 (C-9), 103.0 (C-9a), 170.0 (C-10), 56.0 (C-3-OCH3).

### 3.4. Antibacterial Activity and Minimum Bactericidal Concentration (MBC)

MIC was determined by the broth microdilution method, according to the standard reference method [40]. Mueller–Hinton broth (MHB) cultures of the standard Gram-positive and Gram-negative bacterial strains incubated for 24 h at 37 °C were used: *Staphylococcus aureus *(ATCC 25923), *Bacilus subtillis* (ATCC 19659), *Enterococcus faecalis *(ATCC 29212), *Escherichia coli *(ATCC 25922), *Pseudomonas aeruginosa *(ATCC 27853), and *Salmonella setubal* (ATCC 19196). The extract and compounds were dissolved in 25% methanol and water. The initial concentration was 1,000 µg/mL for extract and 500 µg/mL for isolated compounds. These initial volumes were serially diluted two-fold to obtain concentration ranges of 7.8–1,000 µg/mL for the extract and 3.9–500 µg/mL for the isolated compounds. 100 µL of each concentration was placed in a 96-well microplate containing 80 µL of MHB and 20 µL of inoculum standardized at 1.0 × 10^8^ CFU/mL by adjusting the optical density to 0.1 at 620 nm (Pharmacia LKB-Ultrospec III spectrophotometer). Ciprofloxacin (Bayer HealthCare®, concentration ranges of 0.5 × 10^−5^–35 µg/mL) and 25% methanol and water were used as positive and negative controls. The plates were incubated at 37 °C for 24 h. The assay was repeated three times. The MIC of samples was detected following addition (50 µL) of resazurin solution (0.2 mg/mL) and incubated at 37 °C for 30 min. Growth of bacteria changes the blue dye to a pink color. The pink color indicates positive growth, while blue indicates growth inhibition. MIC was defined as the lowest sample concentration that prevented this change and inhibited bacterial growth. To determine MBC, a portion from each well showing antibacterial activity (MIC) was plated on Muller-Hinton agar (MHA) and incubated at 37 °C for 24 h. The lowest concentration yielding no growth after this subculture was defined as the MBC [41].

### 3.5. Antifungal Activity and Minimum Fungicidal Concentration (MFC)

Antifungal activity and MIC were determined according to the standard reference method [42], evaluated against *Candida albicans *(ATCC 18804), *C. krusei* (ATCC 6258), *C. parapsilosis* (ATCC 22019), and *C. tropicalis* (ATCC 750). The extracts and compounds were dissolved in 25% methanol and water. The initial concentration was 1,000 µg/mL for extract and 500 µg/mL for isolated compounds. These initial volume were diluted two-fold to obtain concentration ranges of 7.8–1,000 µg/mL for the extract and 3.9–500 µg/mL for the isolated compounds. 100 µL of each concentration was placed in a 96-well microplate containing 80 µL of RPMI 1640 medium. Each well was inoculated with 20 µL of suspension containing 2.5 × 10^3^ CFU/mL of yeast by adjusting the optical density to 0.8–0.1 at 625 nm. The antifungal agent fluconazole (Pfizer®, concentration ranges of 1–64 µg/mL) and 25% methanol and water were included in the assays as positive and negative controls. The plates were incubated for 48 h at 37 °C. MIC was defined as the lowest sample concentration showing no visible fungal growth after incubation time. The MIC of samples was detected following addition (50 µL) of 2.0% triphenyl-tetrazolium chloride (TTC). Growth of yeast changes the samples to a red color. Three replications were maintained. A portion from each well with antifungal activity was plated on Sabouroud agar for to determination of MFC.

### 3.6. Anti-Helicobacter pylori Activity

Phenolic compounds were tested to detect anti-*H. pylori* (ATCC 43504) activity [43,44]. *H. pylori* was cultured in MHB (with 5% calf serum) at 37 °C for 3–5 days in a microaerobic atmosphere (10% CO_2_ and 98% humidity). Muller-Hinton agar (with 5% calf serum) was used for susceptibility testing. Disks of 6 mm in diameter were punched from a sheet of Whatman filter paper, sterilized, and impregnated with 25 µL of each sample (31,25, 62,5, 125 and 250 µg/mL)or solvent alone. The bacterial inoculum was prepared and adjusted to 10^8^ CFU/mL (corresponding to 0.5 McFarland standards). A sterile cotton swab was dipped into the standardized bacterial suspension and used to inoculate the Muller-Hinton agar plates. The plates were allowed to dry for 3–5 min. After that, all disks were placed in plates and maintained at a distance of at least 15 mm from the plate edges and sufficiently separated from each other to prevent overlapping of inhibition zones. A clarithromycin (Abbott®) disk (15 µg/mL), and 25% methanol and water solution-impregnated disks were used as controls. Fifteen minutes following placement of the disks, the plates of *H. pylori *were incubated at 37 °C for 3–5 days in a microaerobic atmosphere (10% CO_2_ and 98% humidity). They were then examined and the diameter of the inhibition zone measured.

## 4. Conclusions

The antimicrobial activity of methanolic extract of leaves from *L. spiralis* possibly results from the presence of both antibacterial and antifungal compounds. These findings could provide an important contribution in the search for new compounds with antimicrobial activity.
